# Modulation of the initial stages of systemic inflammation by intervention in the NFkB signalling pathway

**DOI:** 10.1080/07853890.2021.1894004

**Published:** 2021-09-28

**Authors:** Ângela Amaro-Leal

**Affiliations:** Faculty of Medicine of Lisbon (FMUL), Cardiovascular Autonomic Function (CAF) Lab of the Cardiovascular Center of the University of Lisbon (CCUL)

## Abstract

Sepsis is a systemic inflammatory state mediated by the innate immune system resulting in an excessive cellular response to severe infection, with high levels of morbidity and mortality. Furthermore, patients who survive sepsis, have long-term cognitive and functional impairment. Animal models are essential to clarify the pathophysiological mechanisms of sepsis. Herein, we characterise an animal model of lipopolysaccharide (LPS)-induced inflammation, evaluating neural and cardiac function, during the initial stages of infection. Male Wistar rats (12–20 wks, *n* = 24) were injected with LPS (E. coli serotype O127:B8; tail vein) and divided into 3 groups: LPS6 (6 mg/kg), LPS12 (12 mg/kg) and Sham (NaCl 0.9%; 0.1 ml/100g). At 6 and 24 h after LPS injection, an autonomic evaluation was performed in both conscious animals, with continuous radio-telemetry recording of blood pressure (BP) and heart rate (HR), and in anaesthetised animals (induction: sodium pentobarbital, 60 mg/kg, ip; maintenance: 20% solution, v/v) with BP, ECG, HR, tracheal pressure, respiratory frequency (RF) and body temperature continuously monitored. Baro and chemoreflex were evaluated with phenylephrine (0.2 ml, 25 µg/ml; iv) and lobeline (0.2 ml, 25 μg/ml; iv), respectively. Behavioural changes were also evaluated through the elevated-plus maze, open-field and Y-maze tests. Immunohistochemistry and RT-PCR were executed to determine heart and brain inflammatory state. Serum biomarkers levels for organ disfunction were also measured. Overall our results show a rise in BP and HR, and elevated RF due to increased chemoreflex sensitivity. 24 h following LPS injection, all groups have a significantly decreased baroreflex (*p* < 0.05), however, 6 h post-injection, LPS12 show a statistically significant increase in baroreceptor reflex (*p* < 0.05). At both time-points, the two LPS groups present an anxiety-like behaviour, associated with less locomotor/exploratory activity and highly significant cognitive impairment (*p* < .0001). The autonomic evaluation of the anaesthetised LPS12 group results in an increase of the autonomic tone at 6 h post-LPS followed by a decrease at 24 h. The LPS6 group shows the opposite profile. Interestingly, conscious animals reveal a slightly different profile in both groups, with a continuous increase in autonomic tone for LPS12 and a decrease 24 h post-LPS in LPS6 group. The molecular studies show reactive astrogliosis and microgliosis, due to inflammatory processes in the hippocampus, as well as, an upregulation of pro-inflammatory factors in the heart and brain. Serum analysis yielded higher levels of biomarkers for renal and liver dysfunction (*p* < .05) and pancreatic and neuromuscular injury (*p* < .05), in the LPS12 group. Concluding, LPS administration induces strong modifications in both cardiac and neurological systems, during the early stages of the systemic inflammatory response. Being a good model for further pathophysiological and pharmacological studies related to Sepsis.

